# ﻿A new electric-blue tarantula species of the genus *Chilobrachys* Karsh, 1892 from Thailand (Araneae, Mygalomorphae, Theraphosidae)

**DOI:** 10.3897/zookeys.1180.106278

**Published:** 2023-09-18

**Authors:** Narin Chomphuphuang, Zongtum Sippawat, Patipan Sriranan, Paveen Piyatrakulchai, Chaowalit Songsangchote

**Affiliations:** 1 Department of Entomology and Plant Pathology, Faculty of Agriculture, Khon Kaen University, Khon Kaen, Thailand; 2 160 village no. 7, Mae Tho, Mueang Tak district, Tak province, Thailand; 3 Department of Biology, Faculty of Science, Khon Kaen University, Khon Kaen, Thailand

**Keywords:** Arboreal theraphosid, habitat, mangrove forests, mountainous, rainforests

## Abstract

The enchanting phenomenon of blue coloration in animals arises from the fact that blue is one of the rarest colors found in nature, and it is a structural color that is produced by the arrangement of biological photonic nanostructures, rather than pigments. This unique coloration has evolved independently in many different species, adding to the fascination and diversity of coloration patterns in the animal kingdom. This study describes a new species of *Chilobrachys* Karsch, 1892 from southern Thailand that exhibits a blue-violet hue resembling the color of electrical sparks. Photographic illustrations, a morphological description, and the natural habitat of the new species are given. The diagnosis, palpal-bulb structures, spermathecae, and stridulatory organ morphology of related species are discussed.

## ﻿Introduction

Tarantulas, captivating creatures belonging to the family Theraphosidae Thorell, 1869, are mainly found in North, Central, and South America, as well as in regions of Europe, Asia, Africa, and Australia (World Spider Catalog 2023). This broad distribution underscores their adaptability and presence in diverse habitats worldwide (Pérez-Miles 2020). The Selenocosmiinae is a subfamily within the Theraphosidae, notable for its high diversity. It is the second largest subfamily of Theraphosidae and is particularly noteworthy for having the largest species distribution in the region spanning from Asia to Australia (Kambas 2023; World Spider Catalog 2023). The Selenocosmiinae possess unique characteristics, including an oval patch of modified setae forming a stridulation organ on their prolateral maxillae (but absent in some genera), a retrolateral cheliceral surface with multiple rows of strikers, mature males lacking a tibial apophysis on their first legs, and posterior sternal sigillae located at a distance from the sternal margins (West et al. 2012). Tarantulas possess a stridulation organ on their prolateral maxillae, consisting of an oval patch of modified setae used to produce sound by rubbing against surfaces, enabling communication with other tarantulas during mating or territorial displays, while they also have specialized rows or patches of modified setae known as “strikers” on their retrolateral cheliceral surface, contributing to the stridulation process; both of these structures are essential for tarantula communication and behavior, particularly in the subfamily Selenocosmiinae (Pérez-Miles et al. 2005; Pérez-Miles and Perafán 2017). The genus *Chilobrachys* Karsh, 1892, which is a diverse group in the subfamily Selenocosmiinae, consists of 31 species that can be distinguished based on well-supported synapomorphies: the stridulating organ comprises strikers with a thorn-like morphology on the chelicerae, and the presence of 1–3 rows of clavate setae (bacillae) (West et al. 2012).

The blue coloration in animals is a fascinating and relatively rare phenomenon in nature, often resulting from the arrangement of biological photonic nanostructures, rather than pigments, and this unique structural coloration has independently evolved in various species, contributing to the captivating diversity of color patterns in the animal kingdom (Prum 1999). Blue color in tarantulas is a unique instance of structural colors that evolved independently at least eight times, and tarantulas display less iridescence and maintain highly conserved reflectance in a specific narrow band of wavelengths, setting them apart from birds and insects (Hsiung et al. 2015).

**Figure 1.**
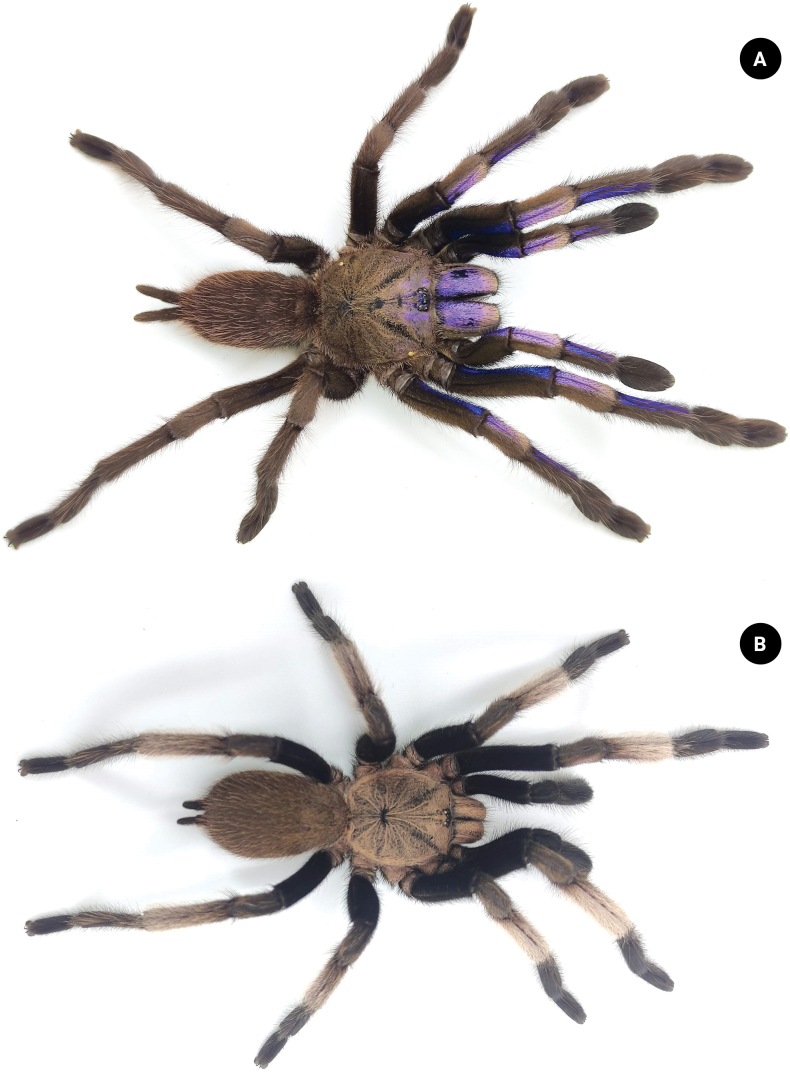
*Chilobrachysnatanicharum* sp. nov. live **A** paratype ♀ ELB03 **B** holotype ♂ THNHM-At-00000062.

**Figure 2.**
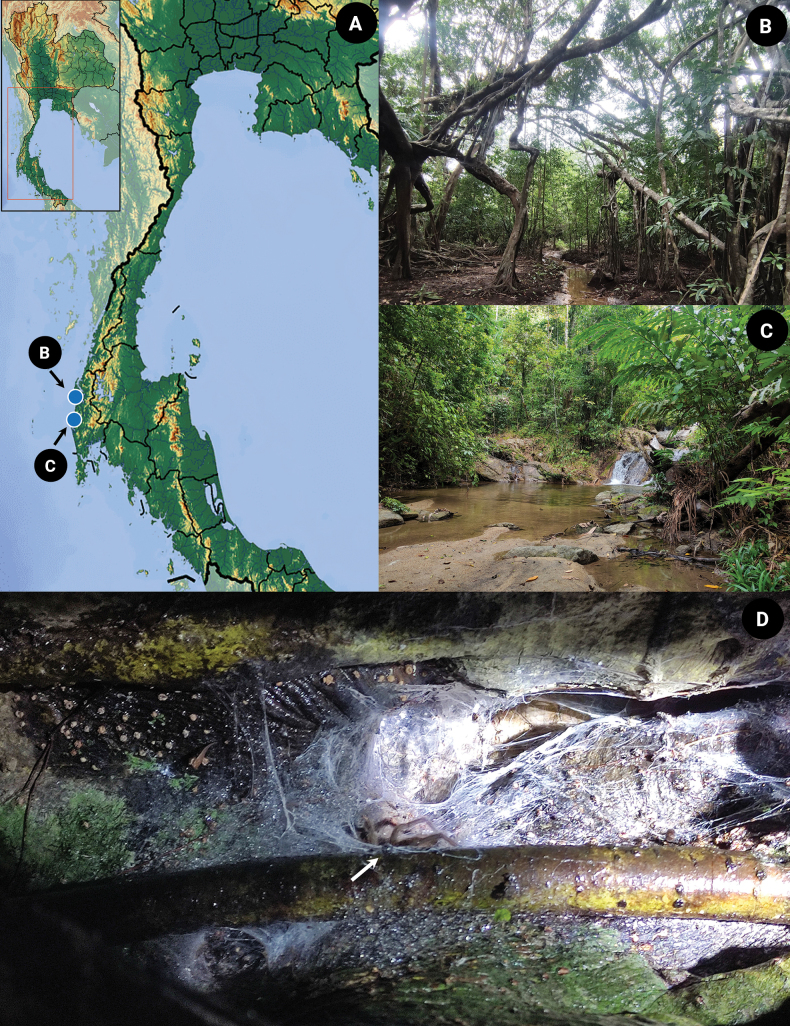
*Chilobrachysnatanicharum* sp. nov. **A** type locality **B** habitat in the type locality of THNHM-At-00000062: Bang Nai Si, Takua Pa District Phang-Nga, elevation 5 m **C** habitat in the type locality of ELB02: Khuekkhak, Takua Pa District Phang-Nga, elevation 57 m **D** juvenile build asymmetric funnel webs live in tree holes.

In this study, we describe a new species of *Chilobrachys* from Thailand that exhibits a blue-violet hue, resembling the color of electrical sparks (Fig. 1), collected from Phang-Nga province, Thailand (Fig. 2).

## ﻿Materials and methods

**Figure 3.**
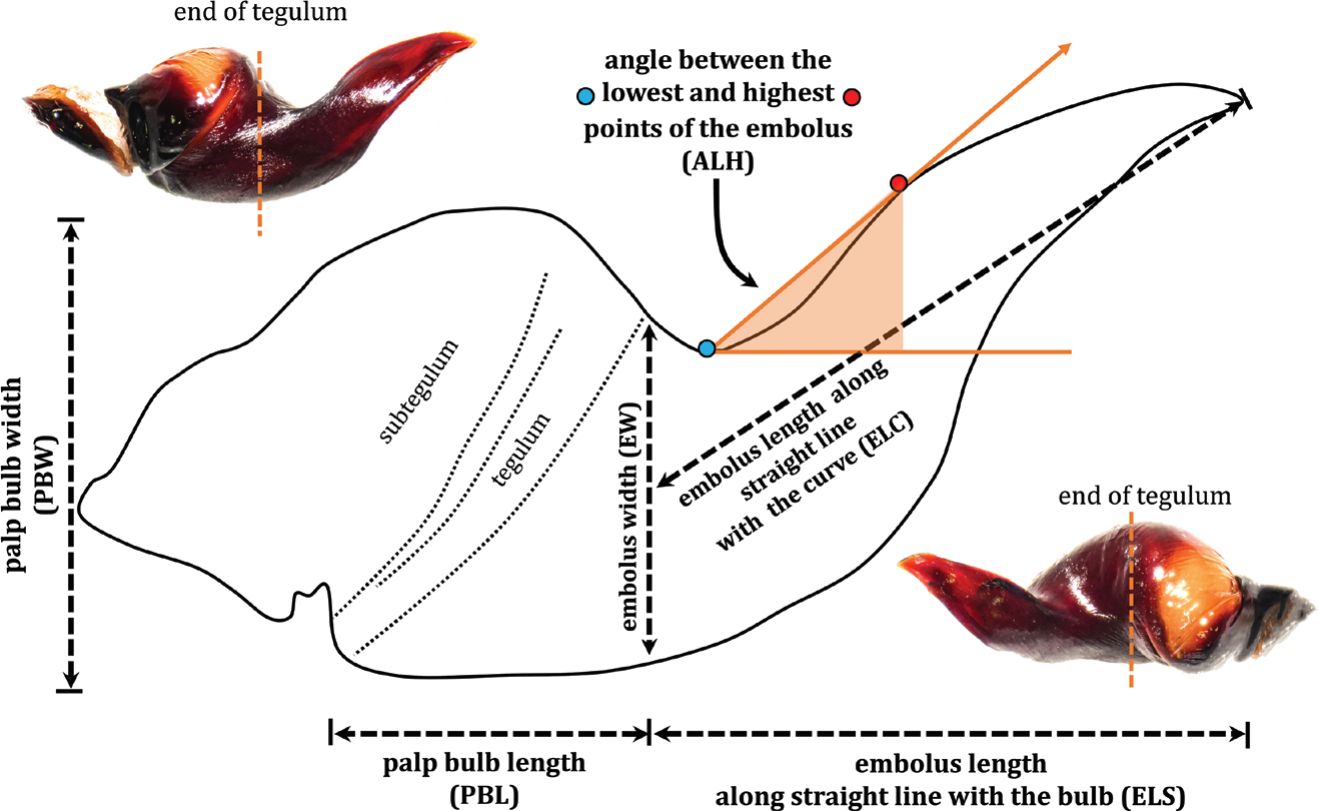
Male palpal bulb measurements and terminology of *Chilobrachysnatanicharum* sp. nov.

The specimens were preserved by fixing them in 80% ethanol and deposited in the Entomology Museum, Faculty of Agriculture, Khon Kaen University (**ENTOKKU**) in Khon Kaen, Thailand and the Natural History Museum of the National Science Museum (**THNHM**) in Pathum Thani, Thailand. The total body length, including cephalothorax, abdomen, and appendage, was measured using digital vernier calipers to two decimal places in millimeters (mm). Measurements were obtained along the central axis of the structures, beginning on the left side, from the mid-proximal articulation point to the mid-distal articulation point, and were recorded according to the method outlined by Hamilton et al. (2016). The measurement process was repeated three times, and the results were averaged. The genitalia and other small anatomical structures were photographed using a digital camera mounted to the phototube of a Nikon SMZ745T or Nikon SMZ25 stereomicroscope. Photographs and counting morphology were taken with the NIS-Elements EDF Module (Extended Depth of Focus) to merge images by selecting the focused regions from each frame. The relation factor (RF) was calculated from the ratio of leg I–leg IV multiplied by 100 (von Wirth and Striffler 2005). The leg formula is also presented, with the leg length in decreasing order. The female genitalia were dissected and then cleared with a solution containing 3 M KOH in solution. The specimens were then compared to those of relatively related species (Zhu and Zhang 2008; West et al. 2012; Nunn et al. 2016; Nanayakkara et al. 2019; Yu et al. 2021; Lin et al. 2022a, 2022b). The terminology for leg spines derives from Petrunkevitch (1925), with revisions suggested by Bertani (2001): **r** = retrolateral, **p** = prolateral, **d** = dorsal, and **v** = ventral. If all spines in the apical region were positioned apically, the term “apical” would be used to describe their position. The terminology used to describe the characteristics of the male palpal bulb and and characteristic groups of keels is based on Bertani (2000) and Gabriel and Sherwood (2019). Based on previous studies, the measurements of palp structures in *Chilobrachys* have been ambiguous. These studies have solely relied on numerical values without delving into the methodology or specific positional measurements. To address this limitation and evaluate the homologous male palpal bulb structures more comprehensively, specific measurement criteria have been provided, particularly concerning the curvature of the embolus (Fig. 3). This standardized approach for measuring the palp will prove beneficial for comprehensively studying the structure of the palp. Moreover, it will enable future characterizations and the identification of diagnostic features.

Male palpal bulb measurements for *Chilobrachysnatanicharum* sp. nov. were conducted using NIS-Element software, and the corresponding terminology is illustrated in Fig. 3. These measurements include:

- **Palp bulb width (PBW)**: Measured from the lowest to the highest point of the bulb.
- **Palp bulb length (PBL)**: Measured from the beginning of the subtegulum to the end of the tegulum.
- **Embolus width (EW)**: Measured from the end of the tegulum along a straight line to ventral of the embolus.
- **Embolus length along a straight line with the bulb (ELS)**: Measured from the end of the bulb length to the tip of the embolus, following a straight line along the length of the bulb.
- **Embolus length along the curve (ELC)**: Measured from the middle of the embolus width to the tip of the embolus, following a straight line along its curve.
- **Angle between the lowest and highest points of the embolus (ALH)**: Measured from the middle of the lowest to the highest point of the dorsal embolus.

These abbreviations are used to describe characters in the text: **Fem** = femur, **Pat** = patella, **Tib** = tibia, **Met** = metatarsus, **Tar** = tarsus; **AME** = anterior median eyes; **PME** = posterior median eyes; **ALE** = anterior lateral eyes; **PLE** = posterior lateral eyes, **PLS** = posterior lateral spinnerets; **PME** = posterior median eyes; **PMS** = posterior median spinnerets; **A** = apical keel **PS**; = prolateral superior keel; **PI** = prolateral inferior keel.

### ﻿Comparative materials examined

*Chilobrachyshuahini* Schmidt & Huber, 1996 Songkhla, Thailand (1 ♀ non-type SPR190304003: ENTOKKU).

*Chilobrachys* sp. Kanchanaburi, Thailand (2 ♂ SPT151434011, SPT151436013: ENTOKKU).

*Cyriopagopusminax* (Thorell, 1897) Chiang Mai, Thailand (1 ♂ non-type T17-HM1: ENTOKKU).

*Cyriopagopuslongipes* (von Wirth & Striffler, 2005) Ubon Ratchathani, Thailand. (1 ♂ non-type 86CLO3821: ENTOKKU).

*Taksinusbambus* Songsangchote et al., 2022. Tak, Thailand (1 ♂ holotype TAK1: ENTOKKU).

*Omothymus* sp. Surat Thani & Chumphon, Thailand (2 ♂ OMS01–02: ENTOKKU).

## ﻿Taxonomy

**Mygalomorphae Pocock, 1892**

**Theraphosidae Thorell, 1869**

**Selenocosmiinae Simon, 1889**

***Chilobrachys* Karsh, 1892**

### ﻿ Chilobrachys natanicharum

Taxon classificationAnimaliaAraneaeTheraphosidae

Chomphuphuang, Sippawat, Sriranan, Piyatrakulchai & Songsangchote sp. nov.

2F65980C-89D2-5B78-8CF6-085FA216DBA5

https://zoobank.org/749F5024-8C8B-4347-8B2D-509A9B521A57

Figs 1 , 2 , 3 , 4 , 5 , 6 , 7 , 8 , 9 , 10 , 11 , 12 , 14A–D

#### Type materials.

***Holotype*** 1 ♂ (THNHM-At-00000062), ***Paratype*** 1 ♀ (THNHM-At-00000063), deposited at THNHM, **Thailand: Phang-Nga**: Bang Nai Si, Takua Pa District, elevation 5 m, 19 Nov. 2022. ***Paratype*** 1 ♂ (ELB02), 2 ♀ (ELB03–04) deposited at ENTOKKU, **Thailand: Phang-Nga**: Khuekkhak, Takua Pa District, elevation 57 m, 20 Nov. 2022.

#### Diagnosis.

*C.natanicharum* sp. nov. was included in *Chilobrachys* based on the maxillary lyra of the stridulation organ comprising 1–3 rows of heavy claviform bacillae (West et al. 2012). The male of *C.natanicharum* sp. nov. can be distinguished from *C.dominus*, *C.hardwickei*, *C.himalayensis*, *C.hubei*, *C.jonitriantisvansickleae*, *C.liboensis*, *C.lubricus*, and *C.qishuoi*, by the wide base of the embolus, which is flat and knife-like in shape (Figs 6–9) (long and slender in all other known congeners). *Chilobrachysnatanicharum* sp. nov. similar to those of *C.dominus*, *C.guangxiensis*, *C.hubei*, *C.jinchengi*, *C.liboensis*, *C.lubricus*, and *C.qishuoi*, in having developed apical (A), prolateral inferior (PI), and prolateral superior keels (PS), but it can be distinguished by the presence of parallel longitudinal keels of PI and PS on the distal spine of the embolus (Fig. 8A, C) (not parallel on the distal spine in other species) and the appearance of a groove at the beginning tip of the apical keel (Fig. 9A, B). The males of *C.natanicharum* sp. nov. can be further distinguished from those of *C.dominus* by the angle between the lowest and highest points of the embolus (ALH), which is 40° (Fig. 14A–D) (compared to 90° in *C.dominus*). Females of *C.natanicharum* sp. nov. resemble *C.fimbriatus* in the form of the spermathecae, which have fused spermathecae. However, they can be distinguished from *C.fimbriatus* by the shape of the spermathecae. In *C.natanicharum* sp. nov., the spermathecae are raised and trapezoidal, with a thick, rounded upper edge (Fig. 12A, B). On the other hand, *C.fimbriatus* has M-shaped spermathecae, characterized by a shallow hump and a middle hollow (see West et al. 2012: fig. 30). Females of this species can be distinguished from several other species, namely *C.assamensis*, *C.dyscolus*, *C.guangxiensis*, *C.hardwickei*, *C.hubei*, *C.huahini*, *C.jonitriantisvansickleae*, *C.khasiensis*, *C.lubricus*, *C.nitelinus*, *C.paviei*, *C.qishuoi*, *C.sericeus*, and *C.stridulans*, by the presence of two separate spermathecae receptacles. *Chilobrachysnatanicharum* sp. nov. differs from all other *Chilobrachys* species, except *C.jonitriantisvansickleae* Nanayakkara, Sumanapala & Kirk, 2019, in that female and male juveniles have a metallic color on the legs, carapace, and chelicerae. The new specieds differs from *C.jonitriantisvansickleae* in terms of color shades. *Chilobrachysnatanicharum* sp. nov. shows a violet-blue metallic color (Fig. 1), whereas *C.jonitriantisvansickleae* has a shade of metallic grayish-turquoise-blue sheen (Nanayakkara et al. 2019: fig. 1a).

#### Etymology.

*Chilobrachysnatanicharum* sp. nov. The specific epithet for this species was provided by Nichada Properties Co., Ltd, Thailand, the winner of the auction campaign for choosing the scientific name of the new species. The name is a combination of the names of Mr Natakorn Changrew and Ms Nichada Changrew, who are company executives. All proceeds from the auction were donated to support the education of Lahu children in Thailand and poor cancer patients. The Lahu people are an indigenous hill tribe in northern Thailand (Musoe) and are known for their vibrant culture and traditional way of life. Unfortunately, many Lahu children are denied access to education due to poverty, leaving them with limited opportunities for their future. The goal is to help change this by providing educational opportunities for Lahu children, giving them a chance to break out of the cycle of poverty. Additionally, cancer remains a significant public health issue globally, affecting millions of people each year. Many cancer patients struggle with financial hardship, which can make accessing quality care even more difficult. We believe that everyone deserves access to quality healthcare, regardless of their financial situation.

#### Description.

**Figure 4.**
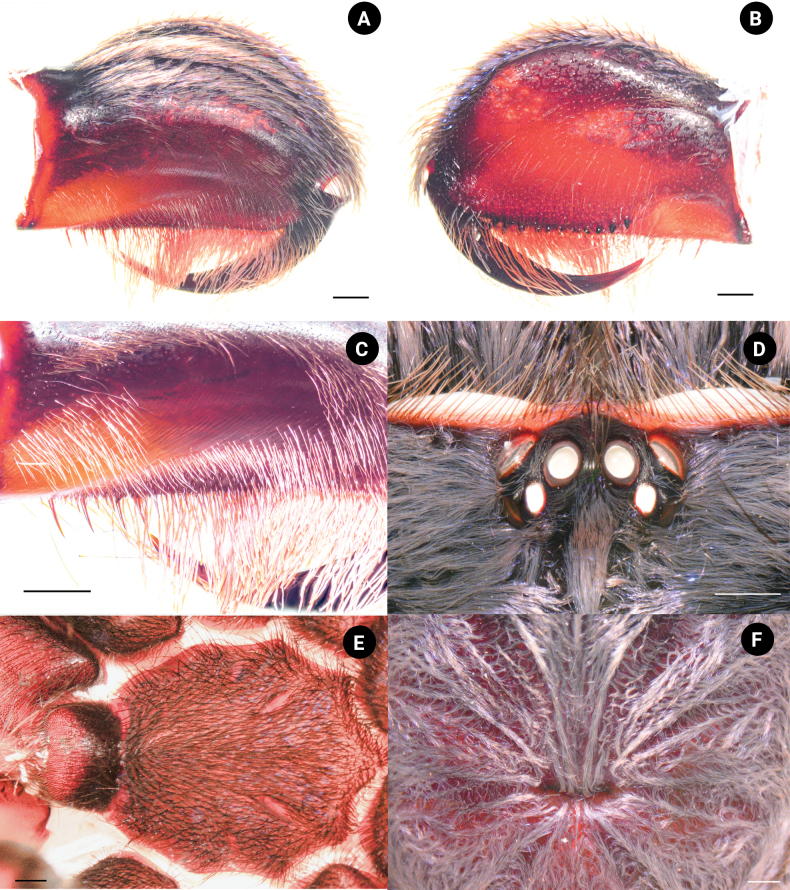
*Chilobrachysnatanicharum* sp. nov. holotype ♂ THNHM-At-00000062 **A** chelicerae, retrolateral view **B** chelicerae, prolateral view **C** chelicerae strikers, retrolateral view **D** ocular tubercle **E** labium, and sternum, ventral view **F** fovea. Scale bars: 1 mm.

**Figure 5.**
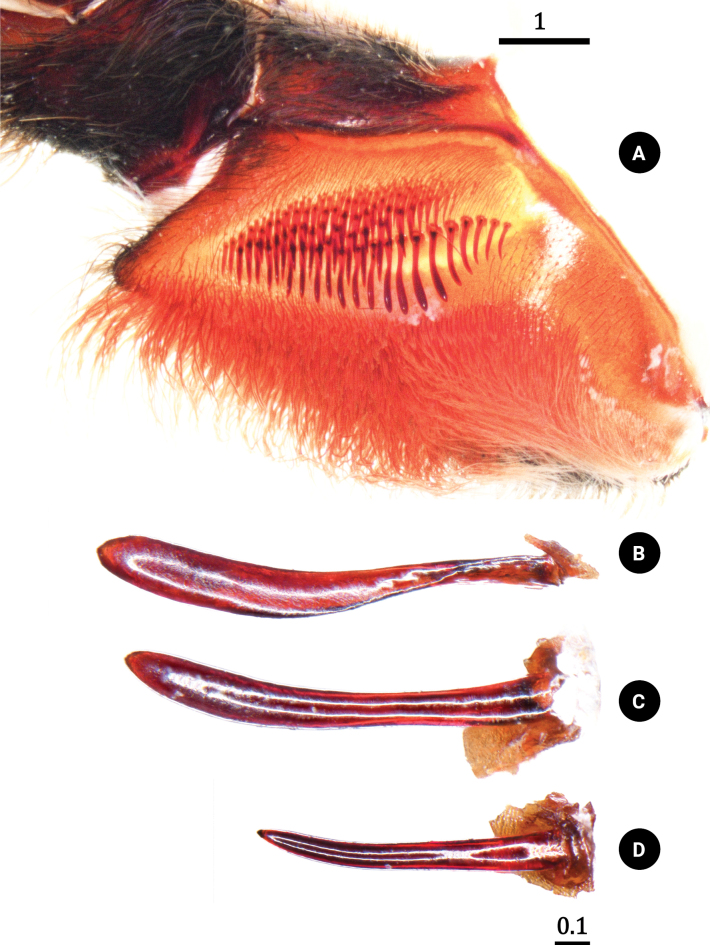
*Chilobrachysnatanicharum* sp. nov. holotype ♂ THNHM-At-00000062 **A** maxilla, prolateral view **B** clavate lyra **C** paddle-like lyra **D** lance-shaped lyra. Scale bars: 1 mm (**A**); 0.1 mm (**B–D**).

**Male. *Holotype*** ♂ THNHM-At-00000062: total length 38.51 (including chelicerae); carapace 14.08 wide, 15.17 long, 4.94 high; procurved deep fovea (Fig. 4F), 2.17 wide; carapace black, covered with short, brownish-gray hairs dorsally and on lateral margins; ocular tubercle (Fig. 4D) 2.74 wide, 1.53 long; clypeus absent. Posterior eye row slightly recurved, anterior eye row slightly procurved; eyes whitish, ALE larger than the round AME; eye size: AME, 0.56; ALE, 0.78; PLE, 0.52; and PME, 0.49. Eye interdistances: PME–PME 1.39; PME–PLE 0.20; PLE–PLE 2.02; ALE–PLE 0.29; ALE–PME 0.38; ALE–ALE 1.74; AME–PME 0.26; AME–AME 0.41; and AME–ALE 0.26; chelicerae dark brown, 8.70 long, 5.87 wide, covered with short, brownish-gray, violet, and metallic-blue hairs dorsally, ventrally covered with long, red-orange setae (Fig. 4A, B), a series of strikers spiniform >4 horizontal rows (Fig. 4C). Maxilla brownish orange, 3.12 wide, 6.65 long, with 275 cuspules, covered with orange-red setae on prolateral surface with the stridulating lyra. The stridulating lyra consists of three types (Fig. 5A): first, a clavate or club-shaped lyra bent and with expanded end (largest part) (Fig. 5B); second, a paddle-like lyra (Fig. 5C); and third, a lyra with a dense covering of lance-shaped setae (Fig. 5D). Labium dark brown, 3.03 wide, 2.04 long, with 634 cuspules. Sternum dark brown, 6.25 wide, 7.34 long, with soft, white hairs and strong, dark hairs, with two pairs of sigillae. Sigilla: anterior pair absent; median pair present, 0.18 wide, 0.56 long, 0.56 from sternal margin of coxa II; posterior pair present, 0.36 wide, 1.04 long, 1.00 from sternal margin of coxa II (Fig. 4E).

**Figure 6.**
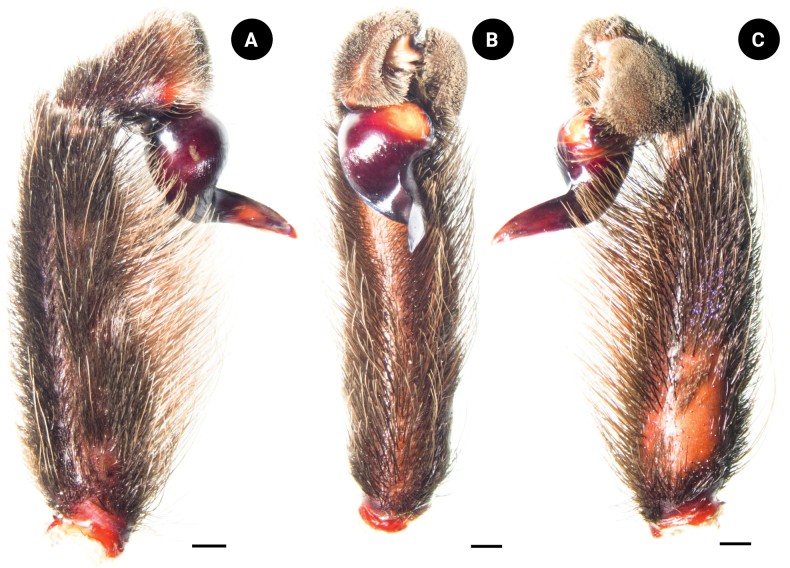
*Chilobrachysnatanicharum* sp. nov. holotype ♂ THNHM-At-00000062 **A** retrolateral view **B** ventral view **C** prolateral view. Scale bars: 3 mm.

***Legs*** dark gray, femur covered with dark hair; prolateral femora I and II covered with violet and metallic-blue hairs. Coxa trochanter and patella dark gray, covered with brownish gray hairs; prolateral patella I and II covered with violet and metallic-blue hairs. Tibia covered with whitish gray hairs; prolateral tibia I and II covered with violet and metallic-blue hairs basally. Metatarsus and tarsus dark gray, covered with short and long, dark-gray hairs (Fig. 1B). The spination (the total number of spines) is expressed for the basal, median, and distal regions on each side: metatarsus I ventral 0–0–1 (apical), metatarsus II ventral 0–0–3 (apical), metatarsus III ventral 0–0–2 (apical), metatarsus IV ventral 0–0–2 (apical), metatarsus IV prolateral 0–0–1. Length of leg and palp segment shown in Table 1. Tibial apophysis absent. Tarsi I–III with two claws and tarsus IV with three claws; two teeth present on claws of tarsi I–III. Scopula undivided on metatarsi II and III, divided on metatarsi I and IV, and divided on tarsi I–IV. Pedipalps dark gray, covered with both long and short, grayish-white hairs on patella and tibia. Pedipalps covered with two hair types: short and dark, and long and brownish gray (Fig. 6A–C).

**Table 1.** Legs and palp measurements (in mm) of holotype ♂ THNHM-At-00000062 *Chilobrachysnatanicharum* sp. nov.; RF = 103, leg formula 1423.

	I	II	III	IV	Palp
**Fem**	18.14	15.53	13.62	17.48	12.4
**Par**	9.29	8.1	7.18	7.65	6.04
**Tib**	17.58	13.94	10.19	15	10.73
**Met**	13.32	11.31	11.64	15.93	—
**Tar**	8.38	7.03	7.47	8.23	3.88
**Total**	66.71	55.91	50.10	64.29	33.05

***Abdomen*** dark brown, 11.46 wide, 17.15 long; abdomen covered with short and long, brownish-gray hirsute dorsally, ventrally, and laterally. Spinnerets dark brown, covered with dark-brown hairs; lateral median spinnerets with one segment 1.60 long; posterior lateral spinnerets with three segments, 7.67 long basal to apical (3.30, + 1.61, + 2.76).

**Figure 7.**
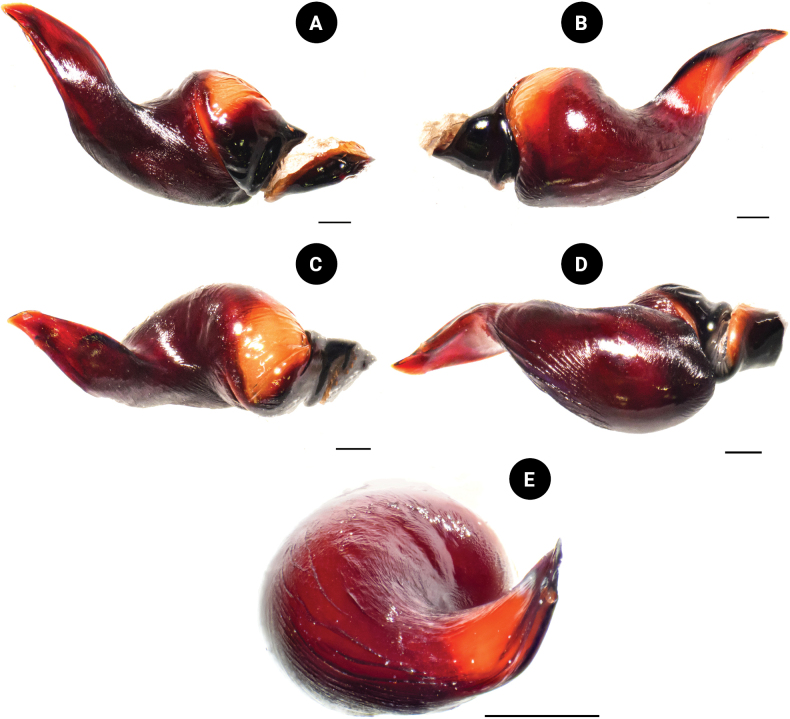
*Chilobrachysnatanicharum* sp. nov. holotype ♂ THNHM-At-00000062, palpal bulb **A** prolateral view **B** retrolateral view **C** dorsal view **D** ventral view **E** apical view. Scale bars: 1 mm.

**Figure 8.**
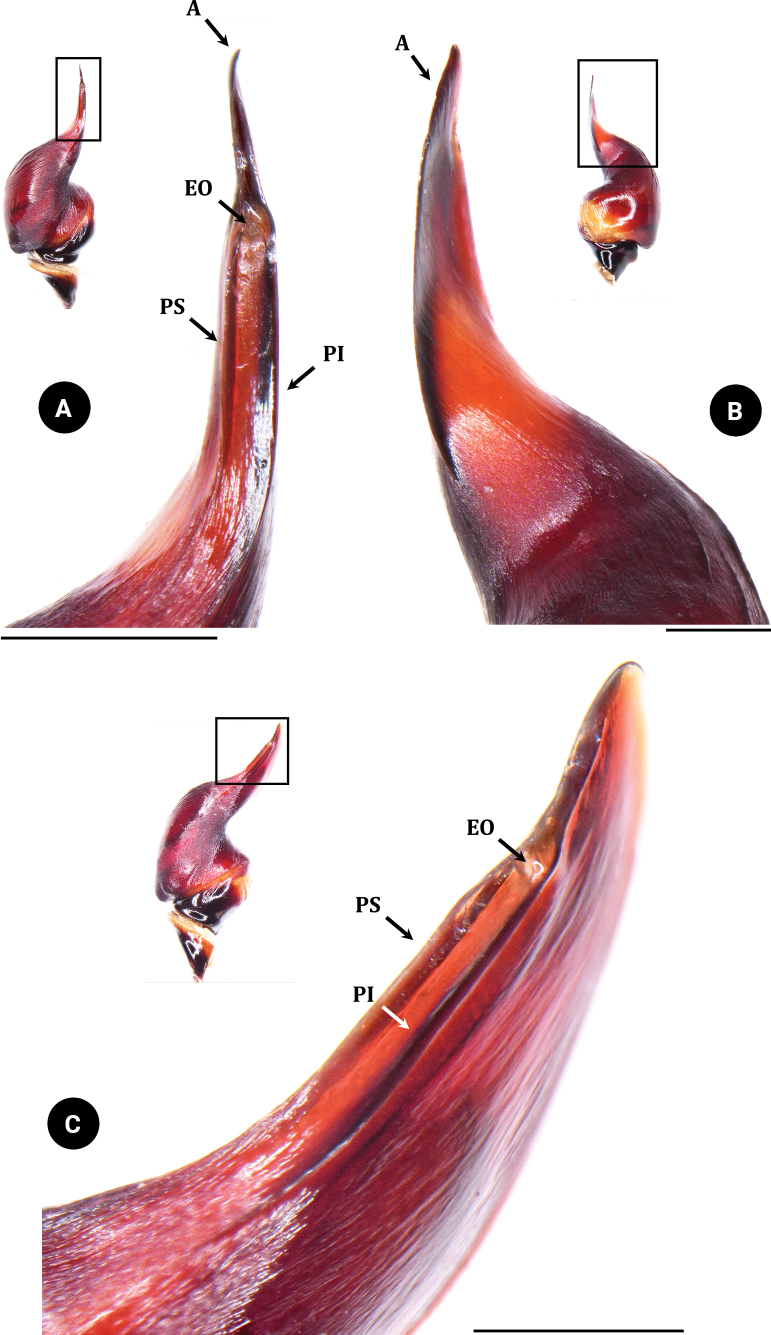
*Chilobrachysnatanicharum* sp. nov. holotype ♂ THNHM-At-00000062, embolus **A** dorsal view **B** ventral view retrolateral view **C** prolateral-dorsal view. Abbreviations: A apical keel; PI prolateral inferior keel; PS prolateral superior keel; EO embolic opening. Scale bars: 1 mm.

**Figure 9.**
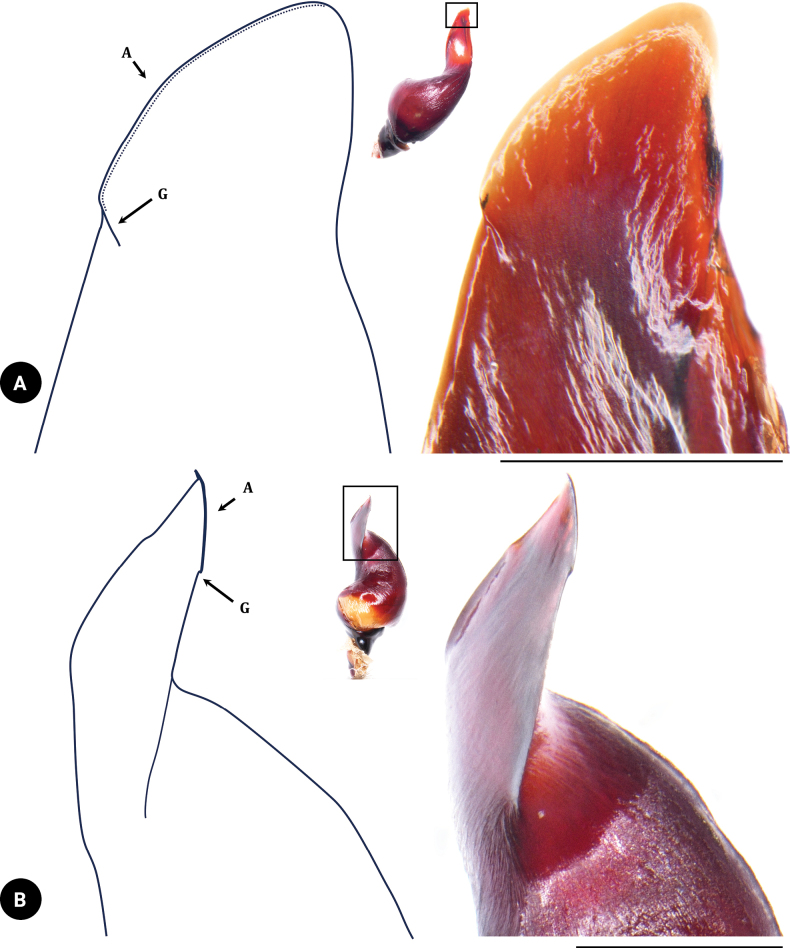
*Chilobrachysnatanicharum* sp. nov. holotype ♂ THNHM-At-00000062, embolus **A** ventral view. **B** dorsal view. Abbreviations: A apical keel; G groove at starting tip of apical keel. Scale bars: 0.5 mm (**A**); 1 mm (**B**).

***Palp bulb and embolus*** (PBL+ELS) 8.02 long, dark reddish brown, palp bulb spherical and partly concave; palp bulb width (PBW) 4.28 and length (PBL) 2.59; embolus width (EW) 3.65 and length along a straight line with the bulb (ELS) 5.43; embolus length along the curve (ELC) 7.10 (Fig. 7A–E). Embolus wide at base and flat, knife-like in shape. Ratios: ELS/PBL = 2.10, ELC/PBL = 2.74, ELC/EW = 1.95, EW/PBL = 1.41, and ELS/EW = 1.49. Palp bulb twisted at 40° angle between lowest and highest points of embolus (ALH) (Fig. 14A, B). Distal spine of embolus with two parallel longitudinal keels, PI and PS (Fig. 8A, C). Spical keel also features a noticeable groove at its starting tip (Fig. 9).

**Male. *Paratype*** ♂ ELB02: total length 55.75 (including chelicerae); carapace 18.14 wide, 20.55 long, 5.29 high; fovea procurved, deep, 2.05 wide; carapace black, covererd weith short, brownish-gray hairs dorsally and on lateral margins, ocular tubercle 2.18 wide, 3.35 long; clypeus absent. Posterior eye row slightly recurved and anterior eye row slightly procurved; eyes whitish; ALE larger than the round AME; eye sizes: AME, 0.56; ALE, 0.85; PLE, 0.59; and PME, 0.52. Eye interdistances: PME–PME 1.74; PME–PLE 0.13; PLE–PLE 2.65; ALE–PLE 0.24; ALE–PME 0.39; ALE–ALE 1.99; AME–PME 0.23; AME–AME 0.57; and AME–ALE 0.29. Chelicerae dark brown, 8.25 wide, 12.27 long, covered with short, brownish-gray, violet, and metallic-blue hairs dorsally; ventrally covered with long, red-orange setae. Maxilla brownish orange, 4.41 wide, 7.97 long, with 337 cuspules, covered with orange-red setae on prolateral surface and with three types of stridulating lyra: clavate, paddle-like, and dense, lance-shaped setae. Labium dark brown, 4.07 wide, 2.47 long, with 687 cuspules. Sternum dark brown, 7.53 wide, 9.60 long, with soft, white hairs and strong, dark hairs, with two pairs of sigillae. Sigilla: anterior pair absent; median pair 0.40 wide, 0.88 long present 0.50 from sternal margin of coxa II; posterior pair 0.36 wide, 0.98 long present 1.21 from sternal margin of coxa II.

**Figure 10.**
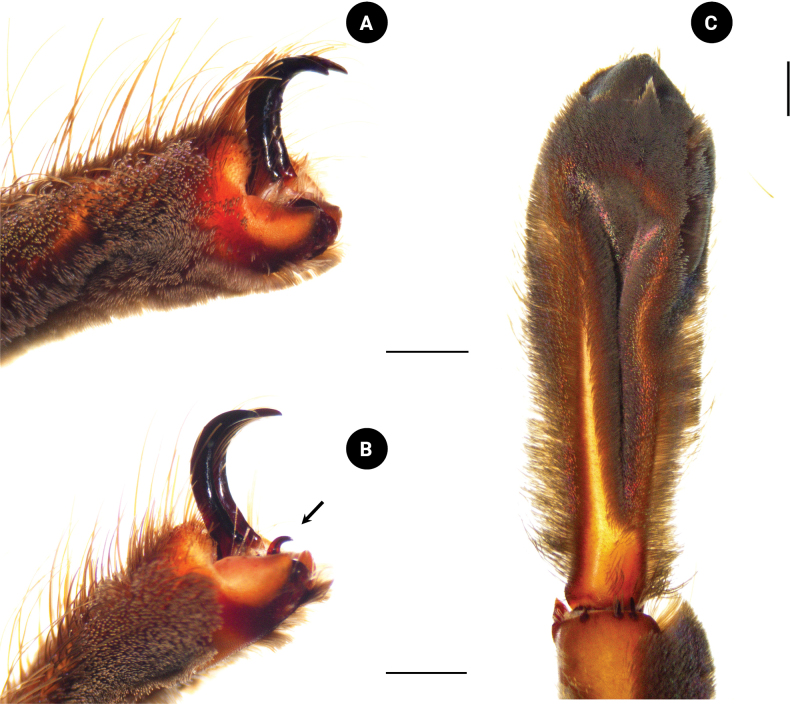
*Chilobrachysnatanicharum* sp. nov. paratypes **A** claws of tarsus II, paratype ♂ ELB02 **B** claws of tarsi IV with third claw, paratype ♂ ELB02 **C** scopula on tarsus I, paratype ♀ ELB03. Scale bars: 1 mm.

***Legs*** dark gray, femur covered with dark hair; prolateral femora I and II covered with violet and metallic-blue hairs. Coxa trochanter and patella dark gray; covered with brownish-gray hairs; prolateral patellae I and II covered with violet and metallic-blue hairs. Tibia covered with whitish-gray hairs; prolateral tibiae I and II covered with violet and metallic-blue hairs basally. Metatarsus and tarsus dark gray; covered with short and long, dark-gray hairs. Spination: metatarsus I ventral 0–0–1 (apical), metatarsus II ventral 0–0–3 (apical), metatarsus III ventral 0–0–2 (apical), metatarsus IV ventral 0–0–2 (apical), metatarsus IV prolateral 0–0–1. Length of leg and palp segments shown in Table 2. Tibial apophysis absent. Tarsi I–III with two claws (Fig. 10A) and tarsus IV with three claws (Fig. 10B), two teeth present on claws on tarsi I–III. Scopula undivided on metatarsi II and III, divided on metatarsi I and IV; divided on tarsi I–IV. Pedipalps dark gray, covered with long and short, grayish-white hairs on patella and tibia. Pedipalps covered with two hair types: short and dark, and long and brownish gray.

**Table 2.** Legs and palp measurements (in mm) of ♂ paratype ELB02 *Chilobrachysnatanicharum* sp. nov. RF = 88, leg formula 4123.

	I	II	III	IV	Palp
**Fem**	22.38	21.62	17.22	24.55	16.49
**Par**	8.89	8.21	7.87	7.57	7.51
**Tib**	21.88	18.29	14.69	20.28	14.25
**Met**	16.40	13.63	15.30	23.54	—
**Tar**	10.36	9.45	9.55	11.07	3.59
**Total**	59.03	52.91	49.94	66.73	27.59

***Abdomen*** dark brown, 16.00 wide, 27.51 long, covered with short and long, brownish-gray hairs dorsally, ventrally, and laterally. Spinnerets dark brown, covered with dark-brown hairs; lateral median spinnerets with one segment, 2.85 long ; posterior lateral spinnerets with three segments 13.84 long basal to apical (4.77, + 3.92, + 5.15).

***Palp bulb and embolus*** (PBL+ELS) 4.03 long, dark reddish brown, palp bulb spherical and partly concave, width (PBW) 2.56, length (PBL) 1.36; embolus width (EW) 2.06, embolus length along a straight line with the bulb (ELS) 2.67, embolus length along the curve (ELC) 4.12. Embolus wide at base and with a flat, knife-like shape. Ratios: ELS/PBL = 1.96, ELC/PBL = 3.03, ELC/EW = 2.00, EW/PBL = 1.51, and ELS/EW = 1.30. Palp bulb twisted at angle of 40° between lowest and highest points of embolus (ALH). Distal spine of embolus presents two parallel longitudinal keels, PI and PS. Apical keel shows a groove at its starting tip.

**Figure 11.**
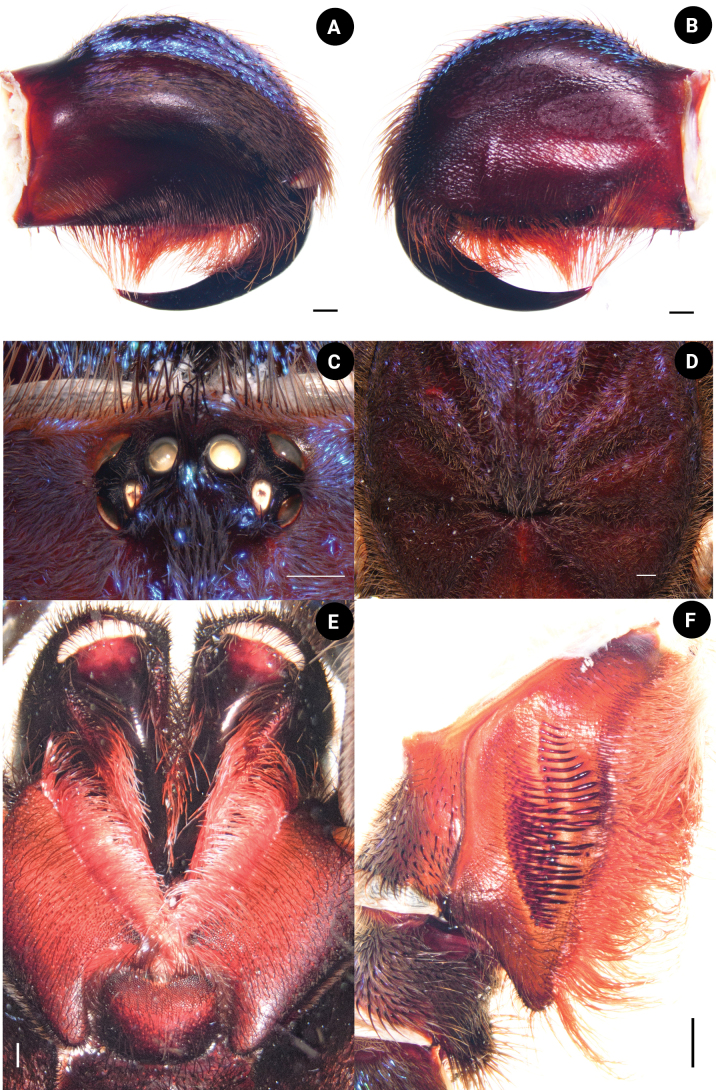
*Chilobrachysnatanicharum* sp. nov. ♀ paratype ELB03 **A** chelicerae, retrolateral view **B** chelicerae, prolateral view **C** ocular tubercle **D** fovea **E** labium and maxilla, ventral view **F** maxilla, prolateral view. Scale bars: 1 mm.

**Female. *Paratype*** ♀ ELB03: total length 56.78 (including chelicerae); carapace 18.09 wide, 21.91 long, 7.98 high; fovea procurved, deep, 2.87 wide; carapace dark brown, covered with short, brownish-gray hairs dorsally and on lateral margins; metallic-blue and violet hairs present on front part of carapace (Fig. 11D). Ocular tubercle 3.35 wide, 2.18 long (Fig. 11C); clypeus absent. Posterior eye row slightly recurved and anterior eye row slightly procurved; eyes whitish, ALE larger than the round AME; eye size: AME, 0.67; ALE, 0.97; PLE, 0.63; and PME, 0.53. Eye interdistances: PME–PME 1.87; PME–PLE 0.22; PLE–PLE 2.72; ALE–PLE 0.34; ALE–PME 0.54; ALE–ALE 2.35; AME–PME 0.41; AME–AME 0.53; and AME–ALE 0.43. Chelicerae dark brown, 8.72 wide, 12.15 long, covered with short, brown hairs, violet and metallic-blue hairs present on dorsally, ventrally covered with long, red-orange setae (Fig. 11A, B). Maxilla brownish orange (Fig. 11E), 4.44 wide, 6.32 long, with 361 cuspules, covered with orange-red setae on prolateral surface with three types of stridulating lyra: clavate, paddle-like, and dense, lance-shaped setae (Fig. 11F). Labium dark brown, 3.79 wide, 2.74 long, with 616 cuspules. Sternum dark brown, 7.81 wide, 8.54 long, with soft, white hairs and strong, dark hairs, with two pairs of sigillae. Sigilla: anterior pair absent; median pair 0.25 wide, 0.70 long present 0.99 from sternal margin of coxa II; posterior pair 0.35 wide, 1.06 long present 1.48 from sternal margin of coxa II.

***Legs*** dark brown, covered with dark-brown hair; prolateral femur, patella, tibiae I and II covered with violet and metallic-blue hairs. Metatarsus and tarsus with violet and metallic-blue hairs absent (Fig. 1A). Spination: metatarsus I ventral 0–0–1 (apical), metatarsus II ventral 0–0–3 (apical), metatarsus III ventral 0–0–2 (apical), metatarsus IV ventral 0–0–2 (apical), metatarsus IV prolateral 0–0–1. Lengths of leg and palp segments shown in Table 3; tarsi I–III with two claws and tarsus IV with three claws, two teeth present on claws of tarsi I–III. Scopula undivided on metatarsi II and III, divided on metatarsi I and IV; divided on tarsi I–IV (Fig. 10C). Pedipalps dark brown, covered with long and short, dark-brown hairs; prolateral femur, patella, and tibia covered with violet and metallic-blue hairs.

**Table 3.** Legs and palp measurements (in mm) of ♀ paratype ELB03 *Chilobrachysnatanicharum* sp. nov. RF = 101, leg formula 1423.

	I	II	III	IV	Palp
**Fem**	20.63	17.46	14.83	18.44	12.87
**Par**	11.59	8.41	7.78	8.49	7.78
**Tib**	15.79	12.75	10.24	14.09	9.00
**Met**	12.69	12.46	12.11	18.22	—
**Tar**	7.66	7.46	5.73	7.86	7.57
**Total**	68.36	58.54	50.69	67.10	37.22

***Abdomen*** dark brown 18.44 wide, 27.72 long, covered with short and long, dark-brown hairs dorsally, ventrally, and laterally. Spinnerets dark brown, covered with dark-brown hairs; lateral median spinnerets with one segment, 3.31 long; posterior lateral spinnerets with three segments 12.75 long, basal to apical (4.94, + 3.45, + 4.36).

The spermathecae are fused and raised, appearing trapezoidal, with a thick, rounded upper edge; basal width 3.85, apical width 2.44, height 1.53.

**Female. *Paratype*** ♀ ELB04: Total length 67.55 (including chelicerae); carapace 24.37 wide, 29.21 long, 12.55 high; fovea procurved, deep, 4.66 wide; carapace dark brown, covered with short, brownish-gray hairs dorsally and on lateral margins; metallic-blue and violet hairs present on front part of carapace. Ocular tubercle 4.22 wide, 2.05 long; clypeus absent. Posterior eye row slightly recurved and anterior eye row slightly procurved; eyes whitish, ALE larger than the round AME; Eye size: AME, 0.69; ALE, 0.90; PLE, 0.76; and PME, 0.74. Eye interdistances: PME–PME 2.09; PME–PLE 0.21; PLE–PLE 3.24; ALE–PLE 0.40; ALE–PME 0.66; ALE–ALE 3.01; AME–PME 0.42; AME–AME 0.56; and AME–ALE 0.62. Chelicerae dark brown, 10.50 wide, 15.50 long, covered with short brown hairs, violet, and metallic-blue hairs present dorsally; ventrally covered with long, red-orange setae. Maxilla brownish orange, 4.88 wide, 11.02 long, with 311 cuspules, covered with orange-red setae on prolateral surface and with three types of stridulating lyra: clavate, paddle-like, and dense lance-shaped setae. Labium dark brown, 4.66 wide, 3.79 long, with 770 cuspules. Sternum dark brown, 9.64 wide, 11.61 long, with soft, white hairs and strong dark hairs, with two pairs of sigillae. Sigilla: anterior pair absent; median pair 0.42 wide, 0.91 long present 1.61 from sternal margin of coxa II; posterior pair 0.53 wide, 1.58 long present 1.61 from sternal margin of coxa II.

***Legs*** dark brown, covered with dark-brown hair; prolateral femur, patella, tibiae I and II covered with violet and metallic-blue hairs. Metatarsus and tarsus violet, metallic-blue hairs absent. Spination: metatarsus I ventral 0–0–1 (apical), metatarsus II ventral 0–0–3 (apical), metatarsus III ventral 0–0–2 (apical), metatarsus IV ventral 0–0–2 (apical), metatarsus IV prolateral 0–0–1. Lengths of leg and palp segments as shown in Table 4; Tarsi I–III with two claws and tarsus IV with three claws, two teeth present on claws of tarsi I–III. Scopula undivided on metatarsi II and III, divided on metatarsi I and IV; divided on tarsi I–IV. Pedipalps dark brown, covered with long and short dark-brown hairs; prolateral femur, patella, and tibia covered with violet and metallic-blue hairs.

**Table 4.** Legs and palp measurements (in mm) of ♀ paratype ELB04 *Chilobrachysnatanicharum* sp. nov. RF = 99, Leg formula 4123.

	I	II	III	IV	Palp
**Fem**	23.90	22.28	17.63	23.02	17.35
**Par**	12.79	12.23	11.20	10.96	9.52
**Tib**	21.16	17.41	11.32	17.01	11.19
**Met**	16.06	15.52	15.38	22.64	—
**Tar**	8.92	9.19	8.29	9.67	9.58
**Total**	82.83	76.63	63.82	83.30	47.64

***Abdomen*** dark brown, 16.81 wide, 30.34 long; covered with short and long dark-brown hairs dorsally, ventrally, and laterally. Spinnerets dark brown, covered with dark-brown hairs; lateral median spinnerets with one segment 3.36 long; posterior lateral spinnerets with three segments 14.23 long, basal to apical (4.55, + 3.71, + 5.97).

**Figure 12.**
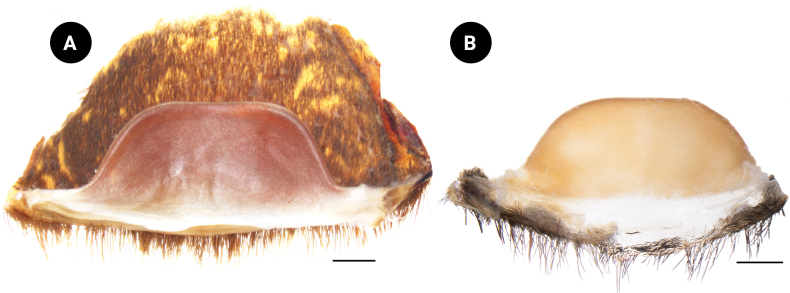
*Chilobrachysnatanicharum* sp. nov. spermathecae, dorsal view **A** ♀paratype ELB04 **B** ♀ paratype THNHM-At-00000063. Scale bars: 1 mm.

The spermathecae are fused and raised, appearing trapezoidal, with a thick, rounded upper edge (Fig. 12A); basal width 8.30, apical width 4.39, height 3.46.

## ﻿Discussion

**Figure 13.**
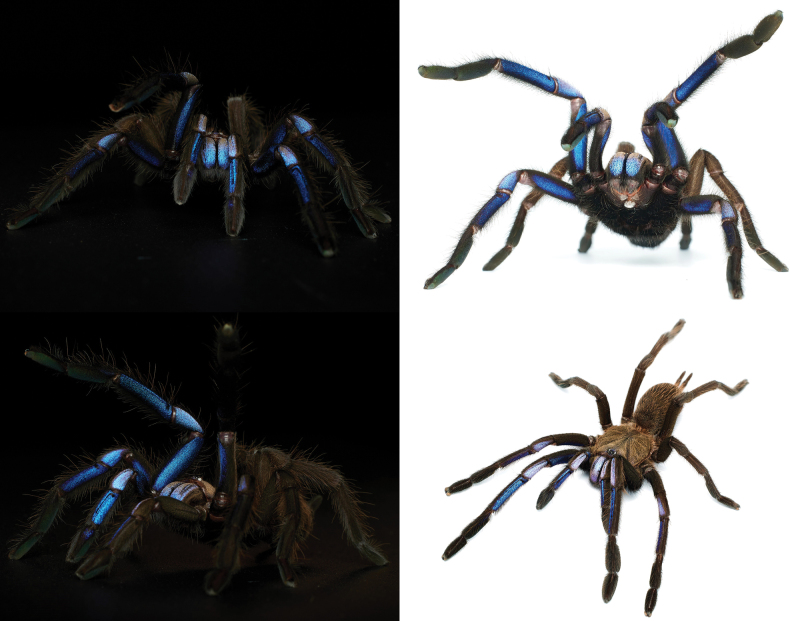
Metallic-blue and violet coloration of *Chilobrachysnatanicharum* sp. nov.

West et al. (2012) proposed the classification of the subfamily Selenocosmiinae and identified three characteristics (synapomorphies) that are shared among spiders in the genus *Chilobrachys*. These features consist of having primary strikers that are thick and sturdy, similar thorn-like strikers on the secondary rows of the chelicera, and 1–3 rows of strong, club-shaped bacillae on the maxillary lyra. The latter feature is a primary taxonomic character for defining genera (Zhu and Zhang 2008; West et al. 2012). Moreover, Gabriel and Sherwood (2019) described the genus *Birupes*, which stands out from all other known Selenocosmiinae due to having the maxillary lyra terminating in a sharp point at their apex. *Chilobrachysnatanicharum* sp. nov. has legs with a unique iridescent blue coloration that is similar to species of Selenocosmiinae such as *Birupessimoroxigorum* Gabriel & Sherwood, 2019 and *Chilobrachysjonitriantisvansicklei*Nanayakkara et al., 2019. However, our research places *C.natanicharum* sp. nov. in *Chilobrachys* due to the presence of club-shaped lyral rows (Figs 5, 11F) that differ from those in *Birupes*, which have lyral rows that terminate in a sharp point (Gabriel and Sherwood 2019: fig. 10). In terms of color, *C.natanicharum* sp. nov. has unique coloration due to the presence of two types of hair: metallic-blue and violet ones (Fig. 13). These hairs are present on various parts of the body, including the chelicera, carapace, and legs. The color depends on the ratio of the two hair colors. Female and juvenile male *C.natanicharum* sp. nov. have more violet than metallic-blue hairs on various body parts, including the chelicera, carapace, patella of the palp, legs I, II, and IV, and tibia IV. Metallic-blue hair is denser on the femora and tibia of the palp and legs I and II. However, on legs III and IV, metallic-blue hair is only present on the femur. In adult male *C.natanicharum* sp. nov., this coloration is also present on the chelicera, carapace, and legs, but it is less intense than in females. Additionally, the color on the tibia changes due to additional dense, white setae.

**Figure 14.**
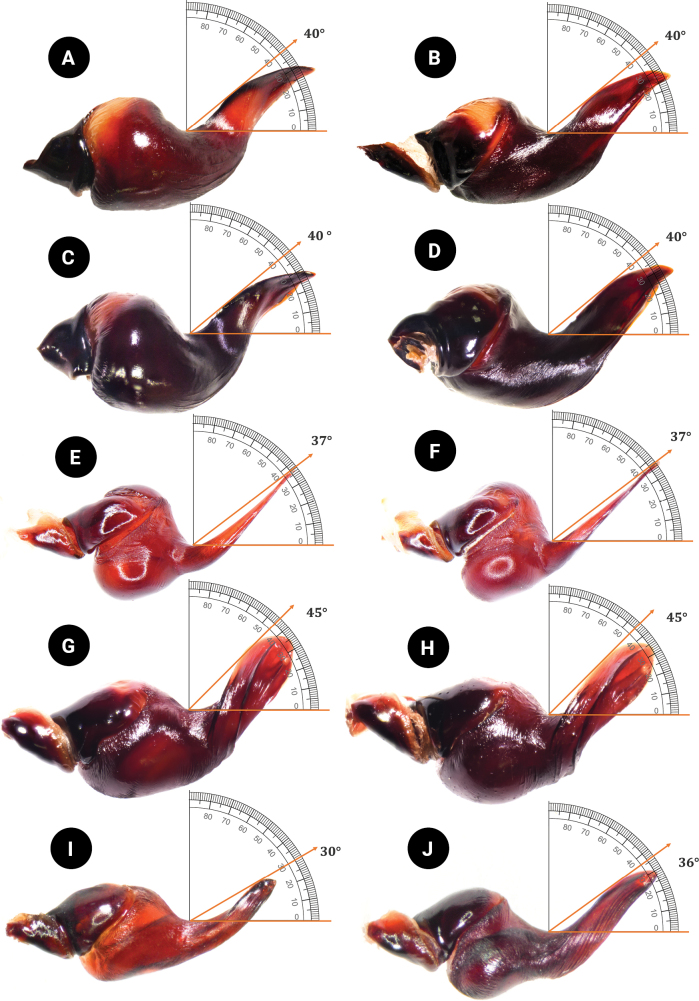
Angle of the lowest to highest point of the embolus (ALH) palpal bulb **A***Chilobrachysnatanicharum* sp. nov. holotype, THNHM-At-00000062, ♂, retrolateral view **B***C.natanicharum* sp. nov. holotype ♂ THNHM-At-00000062, prolateral view **C***C.natanicharum* sp. nov., ELB02 ♂, retrolateral view **D***C.natanicharum* sp. nov., ELB02, prolateral view **E***Chilobrachys* sp., SPT151434011 ♂, prolateral view **F***Chilobrachys* sp. SPT151436013 ♂, prolateral view **G***Cyriopagopusminax* non-type ♂, prolateral view **H***Cyriopagopuslongipes* non-type ♂, prolateral view **I***Taksinusbambus* holotype ♂, prolateral view **J***Omothymus* sp. OMS01 ♂, prolateral view.

We have considered the male palpal bulb character of *C.natanicharum* sp. nov. and have included the criteria to measure the angle from the middle of the lowest to the highest point of the embolus (ALH) in the diagnosis. The results indicate that the angle of projection of the embolus in *C.natanicharum* sp. nov. is 40°, which remains consistent in both retrolateral and prolateral views. This observation was made in two male type specimens (THNHM-At-00000062 and ELB02; Fig. 14A–D). Furthermore, *Chilobrachys* sp. SPT151434011 (Fig. 14E) and *Chilobrachys* sp. SPT151436013 (Fig. 14F), two individuals collected from the same localities in Kanchanaburi, Thailand, also exhibit a similar ALH of 37°. The character ALH can also be applied to other theraphosids, such as *Cyriopagopus*, where both *C.minax* (Thorell, 1897) (Fig. 14G) and *C.longipes* (von Wirth & Striffler, 2005) (Fig. 14H) show a similar ALH of 45°. In contrast, arboreal Ornithoctoninae species exhibit different ALH values, e.g., *Taksinusbambus* Songsangchote et al., 2022 has an ALH of 30° (Fig. 14I), which is lower than *Omothymus* sp. OMS01 at 36° (Fig. 14J). The spermathecae of female *Chilobrachys* spiders are typically bifurcated and consist of twin seminal receptacles. However, *C.natanicharum* sp. nov. is unique among its congenerics, except for *C.fimbriatus* Pocock 1899, because its spermathecae are fused; the new species is distinct from *C.fimbriatus* in the shape of the spermathecae, which are raised in trapezoidal with a thick and rounded upper edge, but M-shaped and with a shallow hump in *C.fimbriatus*. In Selenocosmiinae, such as *Phlogiellus* Pocock, 1897, the presence or reduction of the third claw is a diagnostic character used to distinguish between species (Nunn et al. 2016). However, it cannot be used to distinguish mature specimens of *Chilobrachysdominus* Lin & Li, 2022, *C.jinchengi* Lin & Li, 2022, *C.qishuoi* Lin & Li, 2022, *C.jonitriantisvansickleae*, and *C.natanicharum* sp. nov. (Fig. 10B), as all these species have a third claw present on leg IV.

Previously, the new species of tarantula had been discovered in the commercial tarantula trade market given the name “*Chilobrachys* sp. Electric Blue Tarantula.” However, there was no previous description of the species’ characteristics or natural habitats. The locality of the newly discovered *C.natanicharum* sp. nov. has remained unknown until our recent encounter, leading us to speculate that the species could be distributed in the southern part of Thailand, particularly within the remaining forest patches near the type locality. Our study revealed that *C.natanicharum* sp. nov. inhabits mangrove forests, where tarantulas live inside tree hollows. They can be found at elevations ranging from sea level to highland areas and live in both arboreal and terrestrial burrows within evergreen forests, at elevations of up to 57 m. Unfortunately, the destruction of natural habitats and the hunting of tarantulas have had a devastating impact on local populations in Thailand. As a result, all species of Theraphosidae in Thailand have been listed as controlled wildlife in the Announcement of the Ministry of Natural Resources and Environment on the determination of some wild animals as controlled wildlife for 2022 (https://cites.dnp.go.th). This means that a license is required from the Department of National Parks, Wildlife, and Plant Conservation for the importation or exportation of all tarantulas. It is crucial to conserve these species by protecting their natural habitats through the establishment of protected areas and implementing management plans for both the species and their habitats. Additionally, systematic monitoring is necessary to gather information about their population, and legal breeders should participate in Thai tarantula conservation projects. It is essential to take these steps to prevent further declines in tarantula populations and protect them for future generations.

## Supplementary Material

XML Treatment for Chilobrachys natanicharum

## ﻿Additional information

### Author ORCIDs

Narin Chomphuphuang https://orcid.org/0000-0003-0738-3879

Paveen Piyatrakulchai https://orcid.org/0000-0002-8289-3812

Chaowalit Songsangchote https://orcid.org/0000-0001-7689-5363

### Data availability

All of the data that support the findings of this study are available in the main text.
